# COMMD1 Exemplifies the Power of Inbred Dogs to Dissect Genetic Causes of Rare Copper-Related Disorders

**DOI:** 10.3390/ani11030601

**Published:** 2021-02-25

**Authors:** Ronald Jan Corbee, Louis C. Penning

**Affiliations:** Department of Clinical Sciences, Faculty of Veterinary Medicine, Utrecht University, Yalelaan 108, 3584 CM Utrecht, The Netherlands; r.j.corbee@uu.nl

**Keywords:** COMMD1, copper homeostasis, canine genetics, protein-protein interactions

## Abstract

**Simple Summary:**

Research on rare diseases has specific problems, such as low or small patient groups, limited public awareness, and limited financial support. By definition, a rare disease affects not more than 50 per 100,000 individuals, but with over 6000 unique, rare diseases, more than 300 million people are affected worldwide. Especially, genetic screens are difficult to perform for rare diseases. Due to selective inbreeding in dogs, often these rare diseases present at high frequency in specific dog breeds. This paper in the special issue on “(epi) genetic disorders in companion animals” describes an example of how a novel gene was found that regulates copper accumulation in the liver in a specific dog breed, the Bedlington terriers, and describes an example of how gene products titrate each other’s function on the liver copper accumulation in Labrador retrievers. These two examples clearly show the power in dog genetics for both veterinary and human medicine. Although inbreeding is under great societal scrutiny due to its consequential large number of inherited diseases, dog genetics will directly positively influence animal welfare, in addition to basic knowledge of biochemical regulation systems, and lastly, it will be beneficial for people suffering from rare diseases.

**Abstract:**

Wilson’s Disease is a rare autosomal recessive disorder in humans, often presenting with hepatic copper overload. Finding the genetic cause of a rare disease, especially if it is related to food constituents like the trace element copper, is a Herculean task. This review describes examples of how the unique population structure of in-bred dog strains led to the discovery of a novel gene and two modifier genes involved in inherited copper toxicosis. COMMD1, after the discovery in 2002, was shown to be a highly promiscuous protein involved in copper transport, protein trafficking/degradation, regulation of virus replication, and inflammation. Mutations in the ATP7A and ATP7B proteins in Labrador retrievers and Dobermann dogs resulted in a wide variation in hepatic copper levels in these breeds. To our knowledge, numerous dog breeds with inherited copper toxicosis of unknown genetic origin exist. Therefore, the possibility that men’s best friend will provide new leads in rare copper storage diseases seems realistic.

## 1. Introduction

Copper storage disorders are considered rare diseases. Although the European Union (EU) and the United States have different definitions of rare diseases (EU, not more than 50 per 100,000; US less than 200,000 patients in the US, recalculated as around 86 per 100,000), it is clear that for each individual rare disease, no large patient cohort exists. However, taking into account that around 6000 unique rare diseases are described, the total number of people affected by rare diseases is estimated to be well over 300 million [1]. Almost 75% of rare diseases have a genetic background and are often already present at a young age [2]. People presenting with a rare disease are faced with limited research projects, resulting in superficial biological understanding and treatment options. Moreover, there is a lack of public awareness, or even worse, neglect or the anticipation that it is the patient’s own fault. To address the drawback related to rare diseases, a reference network for rare liver diseases (ERN-RARE-LIVER) was recently established by the European Commission. The Orphanet database provides publicly available epidemiological data on rare diseases (www.orphanet.net (accessed on 2 December 2020)). A rare disease can be rare in the general population but highly prevalent in specific regions. Interestingly a rare disease in men can be, and often is, much more prevalent in in-bred dog strains. 

Wilson disease (WD) and Menkes disease (MD) are rare genetic diseases associated with disturbed copper fluxes in the body. Copper accumulates in the liver in WD patients due to a mutation in the ATP7B gene, which is involved in intracellular copper excretion. In contrast, MD patients suffer from reduced copper levels in various organs, e.g., the brain, due to a mutation in the ATP7A gene, which is responsible for the distribution of copper within the body. The burden on the patient’s quality-of-life is further dependent on their variable clinical presentation. This review paper focuses on the utilization of canine genetics to discover causative and modifier genes of hepatic copper storage diseases in dogs. First, a brief overview is presented on WD and MD, as well as three other very rare copper toxicosis disorders. Second, the unique population structure of dogs will be described to show its feasibility in studying copper-related disorders. Third, examples of a simple Mendelian inherited copper-storage disease (inherited copper toxicosis, Wilson disease alike) and one with a more complex mode of inheritance in specific dog breeds will be presented. Finally, a perspective on how to implement canine patients with a rare disease in (advanced) pre-clinical research to the benefit of a patient with rare copper storage diseases is discussed, although the clinical presentation can be similar but not necessarily identical when comparing these two species.

## 2. Inherited Copper Storage Diseases

### 2.1. Copper Homeostasis

**Figure 1:**
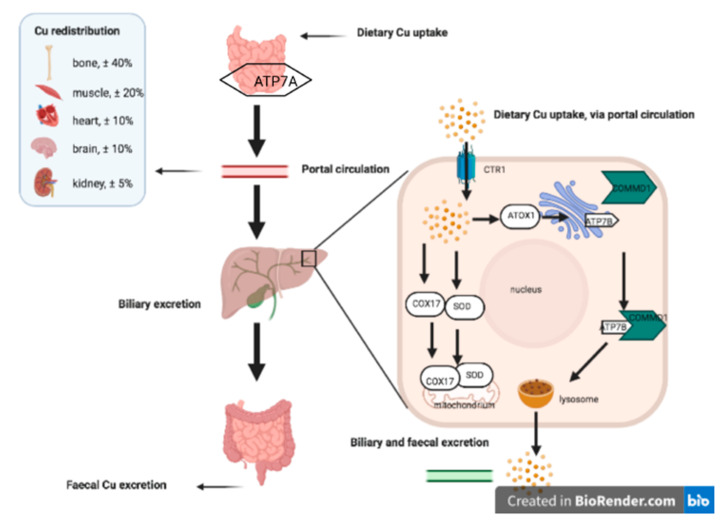
Dietary intake, hepatic homeostasis, and distribution between various organs of copper. Upon dietary intake of copper, ATP7A in the intestines facilitates release into the portal circulation and subsequent uptake in the liver and other organs. Hepatic import is mediated by CTR1 (blue), copper flows along the arrows; either via COX17 and/or SOD into the mitochondria, or via ATOX1 to the Trans Golgi Network (purple). Here, ATP7B interacts with COMMD1 (green) for proper trafficking to the lysosomes (brown), which release ceruloplasmin bound copper the copper for biliary and fecal excretion. Abbreviations: ATOX1, Antioxidant protein1; ATP7A, P-type ATPase 7A; ATP7B, P-type ATPase 7B; CCS, Copper Chaperone for Superoxide Dismutase; COMMD1, COpper Metabolism Murr1 domain-containing protein 1; COX17, Cytochrome c Oxidase Copper Chaperone; CTR1, Copper TRansporter 1; SOD, Superoxide Dismutase.

The trace element copper is like the ancient Roman mythological god of the beginnings, duality, and transitions Janus (or more modern Dr. Jekyll and Mr. Hyde). On the one hand, a good person, and on the other hand, an evil one [3]. Indeed, copper is indispensable for various biochemical processes, yet at the same time, copper is involved in chemical reactions leading to the deleterious production of reactive oxygen species. This is partly due to its transition state (reduced as Cu^+^ and oxidized as Cu^++^). Because of these strongly opposing situations, copper’s intracellular free concentrations need to be kept within very narrow limits [4]. Regulation of intracellular free copper levels occurs at the site of uptake, binding, distribution, and excretion. Copper Transporter 1 (Ctr1) is the main transmembrane copper import molecule, which can also transport zinc [5]. Copper chaperone proteins maintain low intracellular free copper levels and include Cytochrome c Oxidase Copper Chaperone (Cox17), Copper Chaperone for Superoxide Dismutase (CCS), and Antioxidant protein1 (ATOX1) [3,4]. Furthermore, intracellular copper can be sequestered by glutathione, and metallothionein, to minimize its disastrous impact on cellular constituents. The main proteins involved in excretion are the P-type ATPases, ATP7A, and ATP7B [6]. Once excreted, ceruloplasmin mediates copper transport through the bloodstream. Figure 1 depicts the flow of copper from intake to distribution to various organs.

### 2.2. Wilsons Disease

In 1912, Dr. Wilson described 12 patients with neurological presentations caused by an “unknown toxic” compound. It took another 17 years before this “unknown toxic” turned out to be hepatic copper overload, historical review in [7]. The ERN RARE-LIVER database (www.rare-liver.eu (accessed on 30 October 2020)) states Wilson disease as a rare disease, with an estimated clinical prevalence of 1 per 30,000 in the general European population. This is half the prevalence compared to China and several Asian countries [8,9]. For more isolated communities (consanguinity) within Europe, e.g., Sardinia or the Canary Islands, studies indicate a 5–10 fold higher prevalence compared to the general European population [10]. Recent population-based estimates suggest that the genetic prevalence in some areas might be 3 to 4 times the clinical presentation [11], an observation in itself that points to additional factors mediating genotype-phenotype differences. 

In 1993, the causative gene for WD was discovered, being the copper transporter ATPase2 (ATP7B) [12,13]. The organ-specific expression of ATP7B, mainly in the liver but also in other organs such as the brain, kidney, placenta, and parts of the small intestine, explains the clinical hepatic and neurological presentation [14]. Around 50% of WD patients present with a hepatic phenotype, with a 4-times higher female predisposition observed for the severe acute liver failure form. Hepatic presentations are already described in children and young adults. Neurological symptoms occur in a broad range of WD patients (20–65%). The earliest onset of often atypical symptoms, like tremor, gait-related movement symptoms, and or parkinsonism, is at 20–30 years of age [9,14,15]. Characteristic are the Kayser–Fleischer rings due to copper deposition in the cornea, observed in most WD patients [16]. 

The ATP7B protein is responsible for about 95% of the excretion of hepatic copper into the bile. The ATP7B protein consists of eight transmembrane regions forming a membrane-channel, whereas the copper-binding domains are in the N-terminal part within the cytosol. Several established model animals, based on ATP7B mutations, are described to facilitate research. The most frequently studied rodent models include the “Toxic milk” mouse, the TX-J mouse with different mutations in the ATP7B gene, and the ATP7B knockout (ATP7B-/-) mouse [17]. The Long-Evans Cinnamon (LEC) rat has a 900 bp deletion within the 3′- end of the *ATP7B* gene. These rodent models owing to their mutations in the ATP7B gene, do not fully recapitulate the variable clinical presentation of human WD patients [18]. This variation comes with over 500 *ATP7B* mutations (http://www.wilsondisease.med.ualberta.c/databse.asp (accessed on 30 June 2020)) [19,20,21,22]. On top, it suggests the involvement of modifier genes and/or environmental factors that affect the clinical presentation. Amongst these potential modifier genes are *ATOX1*, *XIAP (X-linked inhibitor of apoptosis)*, *MTHFR (5,10-methylenetetrahydrofolate reductase)*, and *COMMD1* (see below for more details on COMMD1, which was first discovered in Bedlington terriers) [23,24,25,26,27]. This emphasizes the need to develop or discover animal models that more closely resemble the subtle modifications in ATP7B function, either caused by the various mutations with the *ATP7B* gene and/or by the activity of modifier genes and/or environmental factors.

### 2.3. Menkes Disease

In contrast to WD, which is associated with copper accumulation in the liver, Menkes disease (MD) is a disease caused by reduced copper levels in various organs. Beginning in the 60′s, the first description of a family with a progressive neurological collapse very early in life was published [28]. This time, sheep grazed on low-copper soil with similar hair (wool-production) defects that linked the neurodegeneration to copper shortage [29]. Especially copper levels in the liver are severely reduced. The lower copper levels obviously affect the copper-dependent enzymes listed above. The birth rate incidence is around 1 in 300,000 in Europe, but in Austria, this figure is 3 to 6-fold higher (www.orphanet.net (accessed on 2 December 2020)). This rare X-linked copper deficiency disorder is caused by mutations in the *ATP7A* gene [30,31,32]. The ATP7A protein is the main excretion pump for copper in the intestine, providing that digested copper is released into the bloodstream and permitting distribution over the various organs. ATP7A and ATP7B share around 50% amino acid homology [33]. As for ATP7B, ATP7A is an eight-transmembrane protein (channel function), with six highly conserved metal-binding domains facing the cytosol. Over 200 different mutations have been reported, but, again, similar to WD, no clear genotype-phenotype correlation has been established [34,35,36,37,38]. The variations in clinical presentation are most likely caused by differential activities of the various copper-dependent enzymes, which urges the need to find innovative genetic screens to dissect how. The low patient number clearly hampers large genome-wide association studies (GWAS), thus it remains to be seen if inbred dog populations can be of help here.

### 2.4. Very Rare Copper Related Diseases

Indian Childhood Cirrhosis (ICC), Endemic Tyrolean Infantile Cirrhosis (ETIC), or Idiopathic copper toxicosis (ICT) are very rare copper overload diseases related to copper homeostasis [39,40,41]. In neither of these diseases, the genetic cause is known, and the obvious candidates like mutations in the *ATP7A*, *ATP7B*, or *COMMD1* (COpper Metabolism Murr1 domain-containing protein 1) genes seem not to be involved [42,43,44]. Additional copper-related disorders, Occipital horn syndrome (OHS), and X-linked distal hereditary motor neuropathy, both related to MD because of ATP7A mutations, and the MENDNIK syndrome (Mental retardation, Enteropathy, Deafness, peripheral Neuropathy, Ichthyosis, and Keratoderma), are reviewed elsewhere [45].

Generally speaking, copper-related disorders are rare to very rare, which in itself comes with specific research limitations, as described in the introduction. The failed wool production in sheep and the inherited copper toxicosis in Bedlington terriers are examples of the benefits to carefully observe diseases in animals. Below, we will move away from human diseases and focus on non-genetically modified animals, more specifically on dogs, as genetic powerhouses to dissect simple and complex genetic diseases.

## 3. Two Decades of Canine Genetics

At present, the OMIA database (www.omia.org (accessed on 2 December 2020)) lists almost 800 traits or disorders described in dogs, compared to around 400 for cats. The initiatives for more concerted investigations of the dog genome were first described in a “briefings” in Science in 1990 entitled Canine Genome Project (doi:10.1126/science.248.4960.1184). In 1993, the Dog Genome Project, founded by Rine and Ostrander, appreciated the value of canine genetics for cancer research [46]. Development of radiation hybrid mapping, comparative chromosome maps, and microsatellite studies, facilitated canine genetic research historical reviews [47,48,49]. The landmark paper by Lindblad-Toh described a draft sequence covering ±99% of the dog genome combined with a single nucleotide polymorphism (SNP) map across 11 breeds [50]. In the mean-time, CanFam1.0 has been updated several times. At present, we are at CanFam3.1. The European Union realized the potential of canine genetics for human genetics and, therefore, the LUPA-consortium was created [51]. Apart from disease-specific discoveries, this consortium focused on 44 genomic regions with extreme variations between breeds [52]. More in-depth sequencing (CanFam 3.1) and RNA Seq data sets from 10 different tissues revealed over 175,000 expressed loci, 21,000 coding loci, in addition, 4600 antisense transcripts, and over 7000 non-coding transcripts [53]. One of the last more generalized (not for a specific disease) discoveries was a set of canine-specific microRNAs based on CanFam3.1 [54]. In order to aid in the annotation of long non-coding RNA sequences, FEELnc, was developed by several LUPA Consortium members [55]. The recently established Dog10K Consortium (www.dog10Kgenomes.org (accessed on 5 January 2021)) aims to describe the enormous phenotypical variation between dog breeds in molecular terms [56]. To that, they target to generate a 20-time coverage of 10,000 dogs.

Within two decades, “the DNA” of dogs has been sequenced and analyzed in extreme depth, together with the development of advanced molecular tools (e.g., 175 kSNParrays, canine-specific micro-arrays), life became a lot easier for researchers in canine genetics and molecular cell biology. In the next paragraphs, inherited copper toxicosis is used to exemplify the potential of canine genetics for human biomedicine.

## 4. The Unique Population Structure of Dog Breeds Amplifies Rare Mutations

Some of the drawbacks related to rare diseases can be tackled by using dogs (*Canis lupus familiaris*) as a genetic magnifying glass. What makes dogs, especially for inherited copper toxicosis, so well-suited [57]? Dogs have been bred and selected for behavioral traits and/or specific morphological features [58,59]. Consequently, this severe artificial breeding pressure resulted in isolated genetic populations of dog breeds with limited genetic variation [58,59,60]. Unintended together with the selection for specific traits, such as excessive muscle formation, short limbs, or a specific coat color, an increased risk for the development of specific disorders with a simple and/or complex inheritance pattern arose within dog breeds. Whereas the genetic variation over the various breeds remained intact, the reduced genetic variability within breeds works as a genetic dissection microscope [59]. Exploiting the downside of inbreeding may, therefore, be instrumental for the discovery of causative and modifier genes involved in rare inherited disorders such as copper storage diseases. For simple Mendelian modes of inheritance, the relatively high number of dogs with a specific disease within a breed is often higher. On top, it is of great importance that the activity of modifier genes within specific breeds is much higher compared to the general dog population and the genetically variable human population. It is especially for complex modes of inheritance that genetic research in dogs is an innovative and powerful approach to aid in human genetic research.

Veterinarians were aware of copper disorders in dogs and sheep for decades [61]. The increased levels of hepatic copper are described in a number of dog breeds, including Bedlington terriers, Skye terriers, West-Highland White terriers, Dobermanns, Dalmatians, and Labrador retrievers [62,63,64,65,66,67]. Often this copper-mediated hepatitis is passed over to the next generation by a complex mode of inheritance, as pedigree studies revealed in most breeds, except the Bedlington terrier. Complex inheritance means that the phenotypic presentation is not dependent on one genetic mutation only but also on environmental factors and/or additional genes.

## 5. Inherited Copper Storage Diseases Are NOT Rare in Dogs

### 5.1. COMMD1 Mutations in Bedlington Terriers

The first description of copper toxicosis in Bedlington terriers was reported in 1979 [65]. In 1999, 20 years later, a genetic mapping study proved that the copper toxicosis locus in Bedlington terriers was located on chromosome 10 region 2p26 [68]. Positional cloning identified, 3 years later, an almost 40kB large deletion covering exon-2 of the *murr1* gene as the causative mutation of Bedlington terrier copper toxicosis [69]. The precise breakpoints in the DNA were described only 3 years thereafter [70]. The current name for MURR1 is COMMD1 (COpper Metabolism Murr1 domain-containing protein 1), which is more in line with the mechanism-of-action of COMMD1 in hepatic copper homeostasis. A few studies proved unequivocally that COMMD1 activity reduces hepatic copper levels [71,72,73,74]. siRNA-mediated *commd1*-gene silencing in HEK293 cells (human embryonic kidney cells) and in BDE-cells (canine liver cells) resulted in elevated intracellular copper levels, even in short-term cultures [71,72]. Liver-specific COMMD1 knockout mice had moderate levels of hepatic copper accumulation, although by no means as high as in the Bedlington terrier dogs [73,75,76]. In liver organoids cultured from COMMD1-deficient dogs, lentiviral reconstitution of a functional COMMD1 protein resulted in a normalization of intracellular copper levels and survival of these cells under high copper culture conditions [74]. These were solid proofs to verify COMMD1s role in copper homeostasis. In addition to direct copper-binding within the COMMD1 protein, it turned out that COMMD1 interacts with ATP7A and ATP7B to facilitate intracellular trafficking of the copper exporting ATPases from the Trans-Golgi-Network to the plasma membrane [77,78,79].

Longitudinal studies on COMMD1-deficient dogs revealed a progressive development of hepatitis similar to the development of chronic hepatitis in men [80,81], yet transplantation of autologous liver organoids with a functional COMMD1 protein did not restore copper excretion in COMMD1 deficient dogs [82].

**Figure 2:**
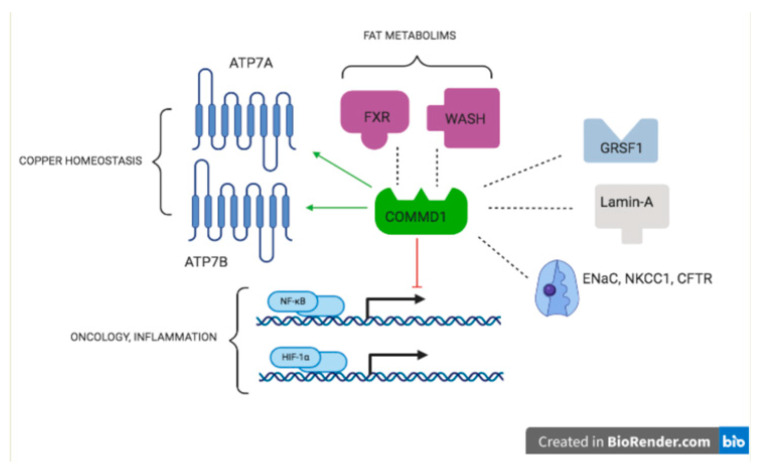
COMMD1 works as a Central Organizing Molecule in Metabolism and Disease. Interactions of COMMD1 protein with other proteins explain its role in copper homeostasis, inflammation, fat metabolism, and some hallmarks of cancer. Dashed lines indicate an interaction, an arrow indicates an activation, blunted line indicates an inhibition. For details on the interactions with ATP7A/ATP7B see [77,78,79], ENaC [89], NKCC1 [12], CFTR [92], WASH [100,101], NFκB [71,96,97], FXR [102,103,104], lamin A [105], GRSF1 [106], and HIF-1α [98]. Abbreviations: CFTR, Cystic Fibrosis Transmembrane Conductance Regulator; ENaC, Epithelial Sodium Channel; FXR, Farnesoid X-activated Receptor; GRSF1, guanine-rich RNA sequence binding factor 1; HIF-1α, Hypoxia-Induced factor 1α; NFkB, Nuclear Factor kappa-B; NKCC1, Sodium-Potassium-Chloride Transporter 1; WASH, Wiskott-Aldrich syndrome protein and SCAR Homologue.

While investigating its mechanism of action, several un-anticipated functions of COMMD1 were described. This review only briefly addresses the plethora of non-copper transport functions of COMMD1, ranging from protein trafficking/degradation, regulation of virus replication, inflammation, and possible roles in oncology. For a more detailed description of the numerous actions of COMMD1, the readers are referred elsewhere [83,84,85,86,87,88,89]. Yeast-two hybrid screens were used to find COMMD1 interacting protein as a means to dissect its mechanism of action. The first interacting protein described was the delta epithelial sodium channel (ENaC), COMMD1-ENaC interacting reduced the cell surface expression of ENaC [89,90]. In contrast, membrane expression of the Na-K-Cl co-transporter (NKCC1) was enhanced by COMMD1 [91]. The intracellular trafficking of the cystic fibrosis transmembrane conductance regulator (CFTR) was enhanced in a COMMD1-overexpressing in vitro model, although indications that this occurs in vivo are lacking [92]. The involvement of COMMD1 in endosomal sorting of low-density-lipoproteins (LDL)-receptors is confirmed in vivo in mice and Bedlington terriers [93,94]. COMMD1 plays a role in the inhibition of NF-κB activity. It does so by interfering in the ubiquitin-mediated proteolysis of Inhibitory B (IκB) [95,96,97]. COMMD1 has an opposing effect on solid tumors versus lymphomas: In patients with solid tumors, decreased COMMD1 expression was related to metastasis and neovascularization [98], whereas in lymphomas, high COMMD1 expression was correlated with worse prognosis [99]. This promiscuous behavior of COMMD1 is summarized in Figure 2.

### 5.2. ATP7A and ATP7B Mutations in Labrador Retrievers and Dobermann Dogs

Labrador retrievers are among the most popular dog breeds worldwide. To emphasize the power of canine genetics to dissect genetic causes in complex genetic diseases, as little as 235 dogs were enrolled in a canine-specific genome-wide association study (GWAS). This study revealed that an Arg1453Gln substitution in the ATP7B protein was related to increased hepatic copper levels, whereas a Thr327Ile mutation in the ATP7A protein partially rescued the ATP7B phenotype [107]. Similarly, an *ATP7B* mutation in Dutch and USA Dobermanns increased hepatic copper levels. A mutation in ATP7A was also found. However, there were too few cases to draw conclusions for the US cohort [108]. Beauty is in the eye of the beholder, but the fact that *ATP7A* is a modifier gene for *ATP7B* is astonishing, given their high level of homology. In this breed, the high prevalence of mutations in the *ATP7A* gene showed the power of canine genetics to find modifier genes. The fact that these mutations are in a gene that is causative for MD, further highlights the beauty of canine research. It is unlikely that this would have been discovered in men where MD is affecting 1 in 300,000 people. It turned out that COMMD1 mutations were involved in neither Labrador nor Dobermann copper toxicosis [109]. Recently, *RETN* (coding for protein RESISTIN) was discovered as a novel modifier gene in copper toxicosis in Labrador retrievers [110]. RESISTIN is involved in hepatic fat storage and mitochondrial defects, however, its low expression in the liver makes it difficult to directly associated *RETN* mutations with hepatic copper accumulation.

## 6. Dogs and Cats Are Clearly Different with Regard to Hepatic Copper Accumulation

Although less stringent, inbreeding occurs in the feline pet population. Much less is known about the genetic background of hepatic copper accumulation in cats. The first description was in 1995 in Siamese cats [111] (Hayes Wade 1995). Copper-induced chronic hepatitis in a European short was presented a decade later [112] (Meertens 2005). The levels of copper, and some other trace elements in feline livers were described by various groups [113,114,115,116] (Andreani 2010; Whittemore 2012; Passlack 2014; Yamkate 2020). In addition to house cats (*Felis catus*), copper levels in other felidae were measured, including bobcats, tigers, cougars, coyotes, and lions [117,118,119] (Bernard 2015; Thomason 2016), Hough 2020. It was only in the last few years that two publications found mutations in the *ATP7B* gene [120,121] (Asada 2019; Asada 2020). To our knowledge, no cats have been described with mutations in the COMMD1 gene, not even in a survey of over 800 cats from Finland [122].

## 7. The Mutual Benefits for Men and Dogs in Rare Copper Storage Diseases

Copper-related diseases in humans are often rare diseases but account for about one-third of the chronic hepatitis cases in dogs [123]. This review has focused on copper dyshomeostasis and described the potential power that genetic research in dog breeds could have to benefit human biomedicine. Dogs turned out to become powerful model animals for men [18,61,124]. Almost 20 years after the discovery of COMMD1 as a causative protein in inherited copper toxicosis in a specific dog breed, a wealth of novel functions related to COMMD1 were described. This novel gene product was not involved in copper overload diseases with thus far unknown genetic causes, like ICC, ETIC, and ICT. Genetic studies in dogs are promising, thus it is hopeful that for several other dog breeds, the genetic cause for inherited copper toxicosis will be elucidated in the near future. We do not know if this will initiate similar anxiety as did the discovery of COMMD1, but it also provides hope for people suffering from rare copper-related diseases that research tools to help solving the genetic background are just around the corner, or sometimes even closer. Therefore, the European Union (www.lupa.eu (accessed on 1 December 2020)) and the Dutch government provided us with a grant to exploit the downside of genetic inbreeding in dogs to the mutual benefit of men and their best friend. In addition to the likelihood that novel (modifier) genes involved in rare diseases, such as copper accumulation, will be found, this work will also create a genetic base for more realistic large animal models [76,82,124].

Current deep sequencing strategies that are exploited in men will find numerous genetic variants. Whether this will lead to a causality of the novel variants with the phenotype will remain difficult in view of the limited sample size in the case of (very) rare diseases. In addition, taking into account that canine inbreeding is tremendously higher than in humans, much fewer variants will be detected in dogs. The causal association between a novel variant and a specific phenotype will, therefore, be much more easily established. This strategy and the implementation of state-of-art sequencing technology in men and dogs has been nicely described [60].

## 8. Dogs as a Test Bed for Humans with Copper Storage Diseases: Copper-Zinc Interactions

Dogs are ideal for testing nutritional interventions, as, opposed to people, we can keep their dietary intake the same for prolonged periods of time. As already mentioned, copper transporters also transport zinc. By increasing dietary zinc, the absorption of copper will, therefore, be reduced. In dogs, a commercially available diet with low levels of copper (5 mg per kg food, instead of 25 mg per kg in general dog food) and high levels of zinc (250 mg per kg food, instead of 50 mg per kg in general dog food) was able to reduce hepatic copper levels in Labrador retrievers with a history of copper storage disease and kept them clinically healthy up till 43 months after starting dietary treatment [125]. As an alternative, by adding 7.5 mg (twice daily) elemental zinc per kg bodyweight for 3 months, metallothionein is induced, which acts as a chelator for copper both in the intestine as in the liver, which has been demonstrated to reduce copper toxicity in dogs [126]. This could also benefit people with copper storage diseases [127]. While realizing that owners allow their pets access to sweets or to left-overs of their own food, generally speaking, the variation in copper uptake is likely far less than in humans. Food that contains high levels of copper includes liver, hazelnut, Brazil nut (yet low in almond), and cacao-based products. This list clearly shows that the uptake of copper in men will be extremely variable, further limiting clear food-related (intervention) studies. Think twice if you find a dog with copper storage disease.

## Data Availability

No new data were created or analyzed in this study. Data sharing is not applicable to this article.
